# Epidermal Growth Factor Relieves Inflammatory Signals in* Staphylococcus aureus*-Treated Human Epidermal Keratinocytes and Atopic Dermatitis-Like Skin Lesions in Nc/Nga Mice

**DOI:** 10.1155/2018/9439182

**Published:** 2018-05-15

**Authors:** Sun Young Choi, You Jin Lee, Ji Min Kim, Hyun Ji Kang, Sang Hyun Cho, Sung Eun Chang

**Affiliations:** ^1^Department of Dermatology, Asan Medical Center, University of Ulsan College of Medicine, Seoul 05505, Republic of Korea; ^2^Department of Dermatology, Seoul Paik Hospital, Inje University College of Medicine, Seoul, Republic of Korea; ^3^Daewoong Life Science Research Institute, Yongin 17028, Republic of Korea; ^4^Department of Dermatology, Incheon St. Mary's Hospital, College of Medicine, The Catholic University of Korea, Incheon 21431, Republic of Korea

## Abstract

Atopic dermatitis (AD) is a chronic inflammatory skin disease with a defective immunologic barrier, which is aggravated by* Staphylococcus aureus (S. aureus)*. Epidermal growth factor (EGF) suppresses inflammation and EGF receptor inhibitors increased* S. aureus* colonization. Thus, we investigated the potential roles of EGF in AD, which is often aggravated by* S. aureus*. We determined how EGF affects the expression of inflammatory cytokines and antimicrobial peptides (AMPs) in human epidermal keratinocytes (HEKs) treated with heat-inactivated* S. aureus* (HKSA)* in vitro* and 2,4-dinitrochlorobenzene-induced AD-like skin lesions in Nc/Nga mice. HKSA increased IL-6 and NF*κ*B expression; EGF treatment had the opposite effect. EGF increased human *β* defensin-2 expression in HEKs and murine *β* defensin-3 in mice. In mice, both EGF and pimecrolimus groups showed less erythema with significantly reduced inflammation and decreased expression of thymic stromal lymphopoietin. EGF relieved* S. aureus*-induced inflammation and AD-like skin lesions in Nc/Nga mice. Therefore, EGF could be a potential topical treatment for AD.

## 1. Introduction

Atopic dermatitis (AD) is a common chronic inflammatory skin disorder that is characterized by allergic skin inflammation. It is a multifactorial, genetically based disease and has a complex etiology including defective skin barrier function, allergen sensitization, and recurrent skin infections.* Staphylococcus aureus (S. aureus)*, which is the most important Gram-positive microorganism of the normal skin flora, colonizes the skin of most patients with AD. Compared with normal skin, atopic skin is associated with increased adherence of* S. aureus* [1].* S. aureus* infection probably results from a defect in skin barrier function and the loss of certain innate antibacterial activities [2].* S. aureus* strains are part of the human nasal microbiome, and this carrier state has often been associated with type 2 immune responses including AD.* S. aureus* cell walls downregulate the human T cell response to superantigens through a TLR2-dependent, IL-10-mediated mechanism and prevent Th1 cell recruitment [3].

A systematic approach is required for the treatment of AD. Providing general skin care, including patient education, avoidance of irritants or proven allergens, and the use of emollients, is a priority for AD treatment. In addition to systemic treatments, topical agents for both hydration and anti-inflammatory therapy are important for the management of AD. In addition to topical steroids, tacrolimus and pimecrolimus are widely used, but topical treatments remain unsatisfactory.

Epidermal growth factor (EGF), which is secreted by platelets, keratinocytes, and macrophages, plays an important role in wound healing. Epidermal keratinocytes are a rich source of EGF receptor (EGFR) ligands, and EGFR signaling has a major effect on the proliferation and differentiation of keratinocytes. Therefore, EGF plays a key role in skin development and homeostasis [4]. Beyond its role in wound healing and epithelial homeostasis, EGF has a protective function in the epidermal barrier, and AD is characterized by epidermal barrier defects. In one study using an acute AD mouse model, skin transepidermal water loss (TEWL) was significantly attenuated in EGF-treated mice, whereas blocking of EGFR signaling increased TEWL [4]. The study also showed that EGF had an immunomodulatory role in the inflamed skin tissue, showing that EGFR signaling reduces allergen-induced IL-6 production and Th17 responses in the skin. IL-6 is known to prime Th17 differentiation in the skin [4].

In addition to increased barrier permeability, AD is characterized by decreased numbers of antimicrobial peptides (AMPs) and defective innate immune function of the immunologic barrier. Changes in the levels of AMPs are associated with the initiation and development of AD, and human beta defensins are particularly scarce in the lesional AD skin of patients who frequently suffer from bacterial or viral infections [5]. AD is often aggravated by* S. aureus* infection and is frequently improved by the use of antibiotics against* S. aureus*. However, the exact pathogenesis of AD in association with* S. aureus* remains unclear. With regard to another interesting clinical point, it has recently been shown that EGFR inhibitors, which are commonly used anticancer drugs, often increase skin colonization by* S. aureus* [2, 6]. Therefore, we determined whether EGF treatment affects inflammatory signals in* S. aureus-*infected human epidermal keratinocytes (HEKs) and an AD-like mouse model. We investigated the innate immunologic master player, thymic stromal lymphopoietin (TSLP), AMPs, and inflammatory signals in HEKs treated with heat-inactivated* S. aureus* (HKSA)* in vitro*, and in 2,4-dinitrochlorobenzene- (DNCB-) induced AD-like skin lesions in Nc/Nga mice.

## 2. Materials and Methods

### 2.1. Reagents

Heat-killed* S. aureus* was obtained from InvivoGen (San Diego, CA, USA). Recombinant human EGF and pimecrolimus cream (Elidel®) were kindly donated by the Daewoong Pharmaceutical Company (Seoul, Korea). Nicotinamide was purchased from Sigma-Aldrich (St. Louis, MI, USA), and gefitinib was purchased from the AstraZeneca Corporation (San Diego, CA, USA).

### 2.2. Cell Culture

Human epidermal keratinocytes (Thermo Fisher Scientific, Waltham, MA, USA) were cultured in EpiLife medium (Thermo Fisher Scientific) with human keratinocyte growth supplement (HKGS, Thermo Fisher Scientific). Before treatment with the reagents, the cells were cultured in EpiLife medium (Thermo Fisher Scientific) for starvation overnight. The cells were maintained in a humidified atmosphere of 5% CO_2_ at 37°C, and the medium was replaced every 2 days. The keratinocytes were stimulated with 10 MOI HKSA to induce inflammatory cytokines and TLR2 signaling.

### 2.3. Real-Time Quantitative Reverse Transcription-Polymerase Chain Reaction (RT-qPCR)

After treatment, total RNA was extracted from the cells using a ReliaPrep™ RNA cell Miniprep system (Promega, Madison, WI, USA), and 1 *μ*g of total RNA was converted into cDNA using a Takara RNA polymerase chain reaction (PCR) kit v2.1 (Takara Bio Inc. Shiga, Japan), under the following reaction conditions: 45°C for 45 min and 95°C for 5 min. The probes were obtained from Applied Biosystems as Assays-on-Demand Gene Expression Assays (GAPDH: Hs02758991_g1, IL-6: Hs00174131_m1, TSLP: Hs00263639_m1, TLR2: Hs01872448_s1, NF-*κ*B: Hs00765730_m1, and p38*α*: Hs01051152_m1). Reactions were carried out using a ABI StepOne Plus system (Applied Biosystems), and relative transcription levels were determined using* GAPDH* as the reference gene. The data were analyzed using ABI StepOne Plus software (Applied Biosystems).

### 2.4. Enzyme-Linked Immunosorbent Assay (ELISA)

The presence of IL-6 and TLSP at 48 h in supernatants of the treatment reagents or the untreated control was determined using specific ELISA assay kits according to the manufacturer's instructions. The IL-6 and TSLP ELISA kits were obtained from R&D Systems (Minneapolis, MN, USA). After treatment, whole-cell lysates were collected using cell lysis buffer (Cell Signaling Technology, Danvers, MA, USA), and the concentration of TLR2 in the cell lysates was measured using a specific ELISA kit (R&D systems).

### 2.5. In Vivo Assay

Six-week-old male NC/Nga mice were purchased from SaeronBio Inc. (Gyeonggi-do, Korea) and housed in a room maintained at 24°C ± 2°C and 55%  ± 15% humidity, with a 12-h light-dark cycle. A total of 20 mice (5 mice per group) were used in this* in vivo* study. Group 1 was free from AD-like skin lesions (normal control). Groups 2, 3, and 4 had AD-like skin lesions, which were induced using 2,4-dinitrochlorobenzene (DNCB) for 1 week. Group 2 was not treated (positive control). Group 3 was treated with topical pimecrolimus (Elidel) cream (pimecrolimus group). Group 4 was treated with topical EGF solution (1 *μ*g/mL) (EGF group). Topical pimecrolimus cream or EGF solution was applied at 0, 1, 3, 6, and 24 h to the AD-like skin lesions. Skin biopsies were conducted at 1, 3, 6, and 24 h. The dorsal skin tissue was fixed with 4% paraformaldehyde, embedded in paraffin, and sliced into 5-*μ*m sections. Deparaffinized skin sections were stained with hematoxylin and eosin (H&E). The number of inflammatory cells per high-power field (×400) in at least three fields was counted under an optical microscope. Immunohistochemical staining of TSLP, murine beta-defensin-3 (mBD-3), and CD3 was also performed. The intensity of immunostaining was graded as 0 (negative), 1 (weak), 2 (moderate), and 3 (strong).

### 2.6. Statistical Analysis

All experiments were carried out in triplicate and the results are expressed as mean ± standard deviation. *P* values < 0.05 were considered statistically significant. One-way analysis of variance with Dunnett's posttest was performed using GraphPad Prism version 7 (GraphPad Software, San Diego, CA, USA).

## 3. Results

### 3.1. The Expression Levels of IL-6 and TSLP Induced by HKSA Were Downregulated by Recombinant Human Epidermal Growth Factor (rhEGF)

**Figure 1:**
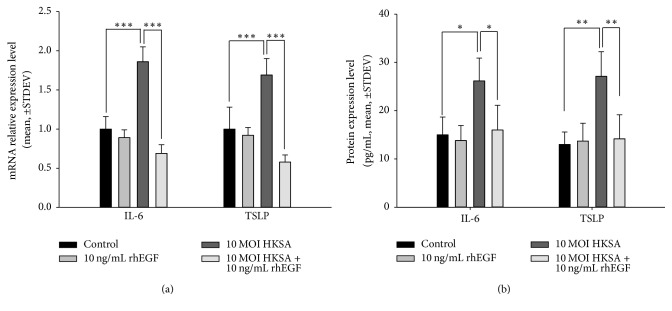
*Recombinant human epidermal growth factor (rhEGF) downregulated IL-6 expression induced by heat-killed S. aureus (HKSA)*. (a) 10 ng/mL rhEGF did not induce IL-6 or TSLP mRNA expression. However, HKSA with a multiplicity of infection (MOI) of 10 (10 MOI HKSA) increased the expression levels of IL-6 and TSLP mRNA; such expression was inhibited by 10 ng/mL of rhEGF. (b) As with the gene results, 10 ng/mL of rhEGF did not affect IL-6 or TSLP protein expression. 10 MOI HKSA induced the expression of IL-6 and TSLP protein; such expression was downregulated by 10 ng/mL of rhEGF. The results are presented as the mean ± STDEV, representative of three separate experiments. Asterisks indicate statistically significant differences (^*∗*^*P* < 0.05, ^*∗∗*^*P* < 0.01, and ^*∗∗∗*^*P* < 0.001).

To investigate our hypothesis, we used HKSA with a multiplicity of infection (MOI) of 10 (10 MOI HKSA) and 10 ng/mL of rhEGF to treat keratinocytes for 6 h and 48 h. We subsequently confirmed the mRNA and protein expression levels of the proinflammatory cytokines IL-6 and TSLP through quantitative reverse transcription polymerase chain reaction (RT-qPCR) and an enzyme-linked immunosorbent assay (ELISA). The mRNA expression levels of IL-6 and TSLP were increased by HKSA and downregulated by rhEGF (Figure 1(a)). Furthermore, rhEGF inhibited the protein expression of IL-6 in a concentration-dependent manner, and the level of TSLP was increased by HKSA (Figure 1(b)).

### 3.2. rhEGF Regulated TLR-2 Expression and Related Signaling in HKSA-Stimulated Keratinocytes

**Figure 2:**
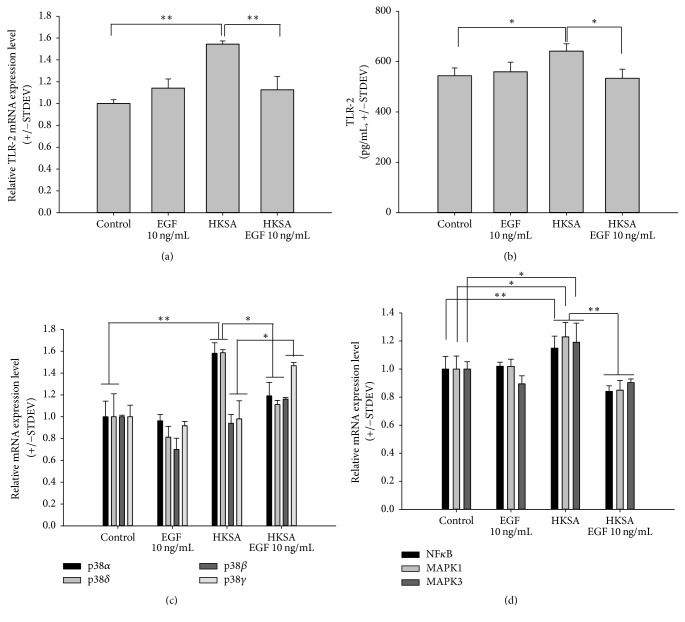
*Recombinant human epidermal growth factor (rhEGF) downregulated the expression of TLR-2 and related signaling markers induced by heat-killed S. aureus (HKSA)*. (a) 10 ng/mL of rhEGF did not induce TLR-2 mRNA expression. However, HKSA with a multiplicity of infection (MOI) of 10 (10 MOI HKSA) increased TLR-2 mRNA expression, which was inhibited by rhEGF 10 ng/mL. (b) Similarly, 10 ng/mL of rhEGF did not affect TLR-2 protein expression. 10 MOI HKSA induced TLR-2 protein expression, which was downregulated by 10 ng/mL of rhEGF. (c) 10 ng/mL of rhEGF did not affect p38 MAPK mRNA expression. However, 10 MOI HKSA increased the expression of p38*α* and p38*δ*; such expression was inhibited by 10 ng/mL of rhEGF. In contrast, 10 MOI HKSA reduced the expression of p38*β* and p38*γ*; such expression was induced by 10 ng/mL of rhEGF. (d) 10 MOI HKSA induced mRNA expression of the TLR-2-related cell signaling markers NF*κ*B and ERK (MAPK1 and MAPK3). Cell signaling mRNA expression was downregulated by 10 ng/mL of rhEGF. The results are presented as the mean ± STDEV, representative of three separate experiments. Asterisks indicate statistically significant differences (^*∗*^*P* < 0.05, ^*∗∗*^*P* < 0.01).

We used RT-qPCR and ELISA to assess TLR-2 gene and protein expression levels, respectively, in keratinocytes when HKSA and 10 ng/mL of rhEGF were added to the culture for 6 h and 48 h. As expected, 10 ng/mL of rhEGF inhibited TLR-2 gene expression in the HKSA-stimulated keratinocytes (Figure 2(a)). Furthermore, TLR-2 protein expression induced by HKSA was inhibited by rhEGF (Figure 2(b)). To determine which transcription factors were responsible for these results, we confirmed mRNA expression of NF-*κ*B, p38 MAPK, and ERK. 10 MOI HKSA increased the expression levels of p38*α* and p38*δ*; such expression was inhibited by 10 ng/mL of rhEGF. In contrast, 10 MOI HKSA decreased p38*β* and p38*γ* expression; such expression was induced by 10 ng/mL of rhEGF (Figure 2(c)). 10 MOI HKSA induced mRNA expression of the TLR-2-related cell signaling markers NF*κ*B and ERK (MAPK1 and MAPK3). Cell signaling mRNA expression was downregulated by 10 ng/mL of rhEGF (Figure 2(d)).

### 3.3. rhEGF Increased Human *β* Defensin-2 (hBD-2) Expression Induced by HKSA but Did Not Increase LL37 Gene Expression

**Figure 3:**
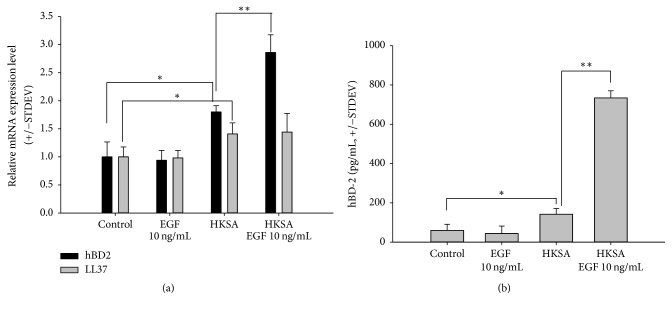
*Recombinant human epidermal growth factor (rhEGF) increased heat-killed S. aureus- (HKSA-) induced hBD-2 expression but did not increase LL37 gene expression*. (a) 10 ng/mL of rhEGF did not induce hBD-2 or LL37 mRNA expression. However, HKSA with a multiplicity of infection (MOI) of 10 (10 MOI HKSA) increased hBD-2 and LL37 mRNA expression. Interestingly, rhEGF and HKSA cotreatment further induced hBD-2 mRNA expression but did not increase LL37 mRNA expression. (b) 10 MOI HKSA induced hBD-2 protein expression. The increase of hBD-2 protein expression was downregulated by 10 ng/mL of rhEGF. The results are presented as the mean ± STDEV, representative of three separate experiments. Asterisks indicate statistically significant differences (^*∗*^*P* < 0.05, ^*∗∗*^*P* < 0.01).

To confirm the effect of rhEGF on the expression of the antimicrobial peptides hBD-2 and LL37, we used RT-qPCR to assess hBD-2 and LL37 gene expression in keratinocytes when HKSA and various concentrations of rhEGF were added to the culture for 6 h. As shown in Figure 3(a), 10 ng/mL of rhEGF did not induce hBD-2 and LL37 mRNA expression. However, 10 MOI HKSA increased hBD-2 and LL37 mRNA expression. Interestingly, rhEGF and HKSA cotreatment further induced hBD-2 mRNA expression but did not increase LL37 mRNA expression. We then used specific ELISA kits to confirm the secretion of hBD-2 in controls that had been treated with HKSA and rhEGF or had not been treated. 10 MOI HKSA induced hBD-2 protein expression. Interestingly, rhEGF and HKSA cotreatment had a synergistic effect on hBD-2 secretion in the keratinocytes (Figure 3(b)).

### 3.4. Topical EGF Reduced Infiltration of Inflammatory Cells in AD-Like Skin Lesions

**Figure 4:**
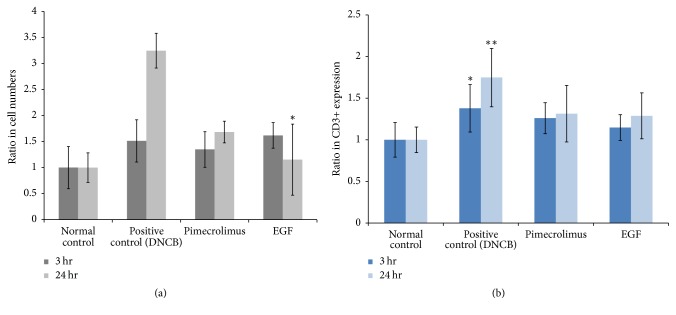
*Topical epidermal growth factor (EGF) reduced the infiltration of inflammatory cells in 2,4-dinitrochlorobenzene- (DNCB-) induced AD-like skin lesions*. (a) The number of inflammatory cells increased in the positive control at 3 h and 24 h. There was reduced infiltration of inflammatory cells in both the EGF and pimecrolimus groups at 3 h and 24 h. (b) CD3 expression increased significantly in the positive group at 3 h and 24 h, and topical pimecrolimus or EGF reduced the level of CD3 expression. The data are represented in graphical form and show the fold changes compared with the normal control. ^*∗*^*P* < 0.05, ^*∗∗*^*P* < 0.01.

We assessed the infiltration of inflammatory cells to determine the effect of topical EGF on inflammation in the AD-like skin lesions of NC/Nga mice. As shown in Figure 4(a), the number of hematoxylin and eosin- (H&E-) stained inflammatory cells increased at 3 and 24 h in the positive control group. However, the application of topical pimecrolimus or EGF reduced the number of inflammatory cells at 3 and 24 h. The reduced infiltration of inflammatory cells was not prominent in the EGF group at 3 h, whereas it was particularly significant at 24 h. As shown in Figure 4(b), CD3 expression increased significantly at 3 and 24 h in the positive control group. As with the results for the number of inflammatory cells, the application of topical pimecrolimus or EGF reduced CD3 expression at 3 and 24 h.

### 3.5. Topical EGF Reduced TSLP Expression and Upregulated Murine *β* Defensin-3 (mBD-3) Expression in AD-Like Skin Lesions

**Figure 5:**
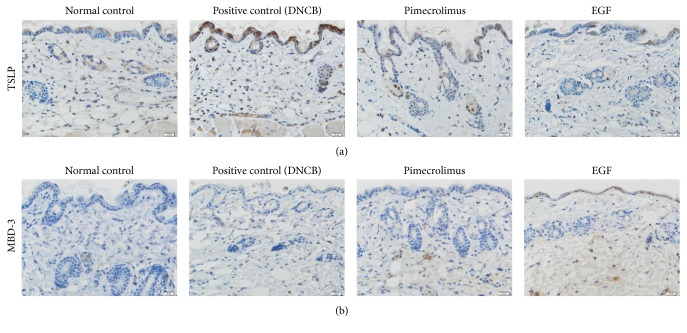
*Topical epidermal growth factor (EGF) reduced thymic stromal lymphopoietin (TSLP) expression and increased mBD-3 expression in 2,4-dinitrochlorobenzene- (DNCB-) induced AD-like skin lesions*. Immunohistochemical staining of TSLP and mBD-3 (high-power field, ×400). (a) There was reduced expression of TSLP in both the EGF and pimecrolimus groups compared with the positive control group at 6 h. TSLP expression was reduced much more in the EGF group than in the pimecrolimus group. (b) mBD-3 expression was induced in the EGF group at 1 h.

We confirmed that TSLP expression was strongly induced at 6 h in DNCB-induced AD-like skin lesions compared with the normal control. However, topical pimecrolimus or EGF reduced DNCB-induced TSLP expression. TSLP expression was reduced much more in the EGF group than in the pimecrolimus group (Figure 5(a)). As shown in Figure 5(b), topical EGF upregulated mBD-3 expression at 1 h; mBD-3 expression was not induced at 1 h in the positive control and pimecrolimus groups.

## 4. Discussion

Previous studies have revealed that EGFR signaling promotes acute wound healing, which involves early recruitment of neutrophils, an increase in the expression of antimicrobial proteins, and ultimately the reestablishment of the physical barrier [7, 8]. Zhang et al. showed that EGFR signaling considerably downregulates the inflammatory cytokines IL-6 and IL-1b after cutaneous allergen exposure by suppressing Th17 cell differentiation [4]. In the current study, we demonstrated that EGFR signaling suppressed* S. aureus*-induced inflammation in human epidermal keratinocytes and the AD-like skin lesions of Nc/Nga mice and also increased the number of AMPs. Furthermore, EGFR signaling showed similar anti-inflammatory efficacy to pimecrolimus, a popular topical immunomodulatory agent used in AD treatment.

TSLP, an IL-7-like cytokine produced by keratinocytes, activates dendritic cells to stimulate naïve T cells to produce T-helper (Th) type 2 cytokines [9]. TSLP promotes Th2 cell responses associated with the pathogenesis of many inflammatory diseases, including atopic dermatitis and asthma, and is highly expressed by keratinocytes in AD lesions [10]. Skin-specific overexpression of TSLP induces AD-like phenotypes in mice, including a dramatic increase in CD4+ Th2 cells expressing cutaneous homing receptors and elevated serum levels of IgE [11]. TSLP is overexpressed in the skin stratum corneum, and its expression correlates with the severity of the AD index score and epidermal barrier function, such as stratum corneum hydration and transepidermal water loss [12]. In fact, TSLP appears to be a key cytokine for the progression of the atopic march (atopic dermatitis to asthma), and treatment with anti-TSLP compounds is expected to have important clinical effects [13]. Our study showed that heat-killed* S. aureus* (HKSA) increased both mRNA and protein expression of TSLP and proinflammatory cytokine IL-6, which is consistent with the clinical finding that AD aggravation can be caused by* S. aureus*. Moreover, EGF (10 ng/mL rhEGF) significantly downregulated the TSLP and HKSA-induced inflammatory signals. Therefore, we suggest that EGF alleviates* S. aureus*-induced inflammatory reactions and further allergic inflammatory responses in AD by regulating the transcription of TSLP. Previous studies have shown that EGFR signaling protects the skin barrier function (e.g., TEWL) following cutaneous allergen exposure [4] and might contribute to the observed suppressive effect of EGF on TSLP, which increased TEWL in the AD skin model.

Next, we focused on TLR-2 because its activation and the production of proinflammatory cytokines such as IL-6 are essential for host defense against* S. aureus* skin colonization in AD. Studies have revealed the relationship between TSLP and TLR-2 during* S. aureus* infection. TSLP transcription is enhanced after stimulation with the wall component of* S. aureus *spp. via TLR2 in canine AD [14].* S. aureus* may contribute to the inflammation of canine AD through a Th2 response via TLR2-mediated TSLP production [14]. Another study has shown that small interfering RNA-mediated knockdown of either TLR2 or TLR6 suppresses* S. aureus* membrane-induced TSLP gene expression [15]. TLR2-deficient mice are highly susceptible to* S. aureus* infection and have a reduced survival rate when infected, which emphasizes the importance of TLR2 in host defense [16]. We demonstrated that EGF reduced the mRNA and protein expression of HKSA-induced TLR-2 in keratinocytes. Furthermore, we confirmed that HKSA-induced TLR2 overexpression in keratinocytes is related to transcription factors, including p38, NF*κ*B, and ERK (MAPK1, MAPK3). In agreement with our study, it has been shown that TLR-2 activation induces IL-6 gene transcription through p38 and NF*κ*B signaling [17]. Previous studies have revealed that EGFR signaling restricts allergen-induced IL-6 production in an acute AD model [4], and EGFR acts as a negative regulator of TLR2 induction via p38 MAPK [18]. Our results indicate that EGF can relieve the inflammatory signals induced by* S. aureus* in keratinocytes by modulating TLR2 and related transcription factors including p38, NF*κ*B, and ERK (MAPK1, MAPK3).

Although skin was formerly considered an inactive physical protective barrier that participates in host immune defense merely by blocking the entry of pathogens, it is now apparent that skin defends the body by rapidly mounting an innate immune response to injuries and microbial insults [19]. AMPs are important in maintaining the innate immune defense mechanism of the skin. The primary defense of the cutaneous innate immune mechanisms is endogenous AMPs secreted by keratinocytes, including hBD2, LL-37, and S100A7 (psoriasin) [20, 21]. In response to infectious microbes such as* S. aureus*, viruses, and fungi, keratinocytes express TLRs and produce proinflammatory cytokines and AMPs (including human *β*-defensin and cathelicidin) [22]. The lesional skin of AD patients shows increased expression of hBD-2 in the stratum corneum, especially during flare-ups of the disease [23]. To date, six hBDs have been identified and characterized in humans. hBD-1 through hBD-4 are primarily detected in the epithelia of the skin, airways, and urogenital tract, whereas hBD-5 and hBD-6 are found exclusively in the epididymis [5]. In general, hBD-1 is regarded as being constitutively expressed, whereas hBD-2 to hBD-4 are commonly inducible following microbial challenge and exposure to proinflammatory stimuli such as lipopolysaccharides, tumor necrosis factor-*α* (TNF-*α*), and interleukin-1*β* (IL-1*β*) [5]. GeneChip microarray analyses demonstrate that AD skin lesions contain lower levels of hBD-2 transcripts compared with psoriasis or normal skin lesions [24]. Actually, it has been demonstrated that AMPs such as hBD-2 and hBD-3 are produced at low levels in the lesional skin of patients with AD relative to patients with psoriasis [5, 22]. Furthermore, hBD-3 is suppressed in AD skin owing to overexpression of Th2 cytokines [24]. A reduced induction of AMPs such as hBD-2 and cathelicidin, in conjunction with defects in the epidermal barrier in AD, contributes to the increased susceptibility of AD skin to* S. aureus* infection [2, 25, 26]. In our study, EGF increased the expression of hBD-2 in HKSA-treated keratinocytes and mBD-3 in AD-like Nc/Nga mice skin lesions, respectively (Figures 3 and 5(b)). According to the gene study, mBD-3 is a homolog of hBD-2 [27]. In mice, this peptide is not constitutive but is inducible against microbial invasion at mucosal surfaces [28]. We observed that EGF alone did not induce hBD-2 (Figure 3(b)), but EGF significantly increased hBD-2 expression in response to* S. aureus*. As in our study, EGF alone did not increase the expression of hBD-2, but when used with IL-1, it increased the expression of hBD-2 in keratinocytes [4, 29]. These results suggest that EGF may play a role in enhancing the immune barrier by cooperating with other cytokines under inflammatory conditions rather than directly inducing hBD-2. We did not observe a change in LL-37 expression following EGF treatment in HKSA cotreatment. This may have been due to the fact that LL-37 is regulated by a mechanism other than TLR ligand-induced HBD-2 expression [30].

Recent studies have shown that EGF significantly reduces IL-6 expression by epidermal keratinocytes through the inhibitory effect of EGF on Th17 cell differentiation [4]. Several researchers have reported the immunomodulatory effect of EGF through the regulation of cytokine and chemokine secretion by keratinocytes, such as the downregulation of the chemokines CCL2, CCL5, CCL27, and CXCL10 [31, 32]. In the current study, EGF reduced the number of inflammatory cells at 3 and 24 h after treatment in AD-like skin lesions (Figure 5). The anti-inflammatory effect of EGF had a similar efficacy to that of pimecrolimus, a topical therapy that is widely used to treat AD. The application of topical pimecrolimus or EGF also decreased CD3 expression at 3 and 24 h (Figure 4). EGF has been used topically to promote wound healing [33], and our data suggest that topical EGF may be beneficial in preventing AD exacerbation. However, despite the demonstrations of efficacy of topical EGF under experimental conditions, further research is needed to determine the efficacy and safety of topical EGF in AD patients. In conclusion, we confirmed that EGF contributed to reducing TLR-mediated inflammatory reactions and promoted AMP production in HKSA-treated keratinocytes and AD-like mouse skin lesions. Considering that AD is characterized by decreased AMPs with defective innate immune function, EGF is expected to be effective in restoring damaged immune barriers by improving defenses such as those provided by AMPs in patients with atopic dermatitis who are vulnerable to* S. aureus*. In addition to the well-known effects of improving the permeability of the epidermal barrier, the immune barrier-modulating function of topical EGF makes it an effective treatment for AD.
